# Provider’s attitudes towards telehealth and parenting interventions during COVID-19 pandemic: an exploratory cross-sectional study from Brazil and Mexico

**DOI:** 10.1186/s41155-025-00360-3

**Published:** 2025-08-04

**Authors:** Marina Kohlsdorf, Cole Hooley, Alejandro L. Vázquez, Mariana M. Juras, Grant Decker, Taylor Iskalis, Kayla Miller, Quinn Tompkins, Nancy G. A. Buenabad, Michela Ribeiro, Acileide C. F. Coelho, Ana A. Baumann

**Affiliations:** 1 Universidade de Brasília, Brasília, Brazil; 2 Brigham Young University, Provo, USA; 3 The University of Tennessee, Knoxville, Knoxville, USA; 4 Florida Institute of Technology, Melbourne, USA; 5 National Institute of Psychiatry Ramon de La Fuente Muniz, Huntsville, Mexico; 6 Aiutare Instituto de Psicologia, Brasília, Brazil; 7 Washington University in St. Louis, St. Louis, USA

**Keywords:** COVID-19 pandemic, Online assistance, Cross cultural research

## Abstract

**Background:**

The COVID-19 pandemic presented challenges for mental health providers all over the world, since they had to abruptly change from in person assistance to remote meetings. The adverse effects from social isolation were critical in Latinx populations such as Brazil and Mexico, since these countries faced a great amount of social, health, and economic burden during the pandemic, which affected families' access to care and increased inappropriate parenting practices.

**Objective:**

This study aimed to understand the impacts of adapting parenting interventions to online sessions for Brazilian and Mexican providers, due to the COVID-19 pandemic.

**Methods:**

Sixty-two Brazilian and 49 Mexican mental health care providers that worked with parenting interventions (including psychologists, social workers, occupational therapists, counselors, and others) took part in this study. The measures included two standardized questionnaires (the Questionnaire about Acceptability, Feasibility and Appropriateness of Telehealth, and the Epidemic-Pandemic Impact Inventory), demographic data, and complementary items developed specially for this study. All measures were translated from English to Brazilian Portuguese and Spanish, resulting in five sets of themes related to (a) service delivery, (b) barriers to mental health assistance, (c) acceptability, feasibility and appropriateness of telehealth, and (d) impacts of pandemic on providers´ professional and personal lives.

**Results:**

For all participants, adapting to online sessions presented challenges related to technology issues, time management, less healthy habits, and overload of chores between work and home tasks, besides concerns related to confidentiality and privacy. Kruskal–Wallis Rank Sum Tests revealed that Mexican providers reported less barriers regarding technology, while Brazilian providers mentioned less economic impact.

**Conclusion:**

This study describes a comparison between providers of two Latinx countries facing demands from COVID19 pandemic, showing common challenges and specific barriers. Suggestions are presented in order to improve the experience of telehealth (i.e., tailored sessions, guidelines for families that ensure privacy, and policies that can increase telehealth access for vulnerable populations).

## Introduction

In December 2019, the World Health Organization (WHO) declared the COVID-19 pandemic a global emergency that demanded lockdowns and social distancing policies, since a total of 695,781,740 cases had been reported until 2024, with 6,919,573 deaths worldwide (WHO, 2024). The COVID-19 pandemic has played a significant role in the disruption of family routines and the quality of family relationships, with an overload of demands placed on caregivers pertaining to the provision of childcare and education (Juras et al., 2024; Marques et al., 2020; Rodrigues de la Iglesia, 2020; Roos et al., 2021).

These adverse effects were critical in Latinx populations such as Brazil and Mexico, since these countries faced a great amount of social, health and economic burden during the pandemic, which affected people's access to care (Araujo & Sarmiento, 2021; Benza & Kessler, 2021). Specifically, the pandemic in these countries jeopardized the families’ healthcare assistance, safety, and economic instability; further psychological effects of the pandemic in both countries included disruption in affective dynamics, daily routines, and parental practices, of which had a crucial impact on both parents´and children's mental health (Benza & Kessler, 2021; Gurgel et al., 2023; Oliveira et al., 2022).

Discernibly**,** the isolating circumstances brought forth by the COVID-19 pandemic have expanded the use of global psychological telehealth services: social distancing, school and service closure, and an increased need for mental health services have yielded a mass crossover to online platforms, particularly for those requiring routine services initiated pre-pandemic (Barnett et al., 2021). Although telehealth has been existent for decades, it was during the pandemic that studies were able to investigate the readiness and feasibility of deploying these initiatives at scale (Boldt et al., 2021; Juras et al., 2024).

Data suggests that telehealth interventions are efficacious methods housed within the provision of web-based family therapy services (Bono, 2022; Domoff et al., 2022; McLean et al., 2021). Client benefits of online therapy include: increase in caregivers´ positive outlook related to their own parental practices, better confidence in their ability to provide care to children, positive views about telehealth, and reduction of parental stress following subsequent improvement in child behavior (Bono, 2022; Domoff et al., 2022).

Some research has explored the perceptions of providers’ attitudes towards telehealth, since the use of online sessions during COVID-19 increased to 85% of their services: they endorsed that online technologies were useful, easy and efficient (McKee et al., 2021; Vázquez et al., 2021). Nonetheless, there are few studies regarding specifically how Brazilian and Mexican mental health providers perceived the transition between in person and online assistance.

In Brazil and in Mexico, providers experienced several challenges shifting to telehealth-based services, as the lack of privacy in their own homes, problems related to Internet connection, security with online data, and the concern about people who could not afford internet and healthcare, due to economic hardship (Caetano et al., 2020; Campos et al., 2022; Carvalho & Mendes, 2023; Messias & Cury, 2021; Santos et al., 2023; Silva et al., 2022; Vidal et al., 2023). There were service interruptions, internet problems, and poor-quality video/audio, which hindered the effective utilization of telehealth services. Furthermore, the lack of technological literacy and support, coupled with a loss of interest among patients, exacerbated the barriers to accessing healthcare remotely. Despite these challenges, telehealth has proven to be viable for both providers and patients (Fonseca et al., 2022; Tiwari et al., 2023).

Research focusing on Brazilian providers showed that self care and physical health were harmed, since the online assistance was perceived as more exhausting and ineffective than in-person psychotherapy (Caetano et al., 2020; Santos et al., 2023). Besides that, there were reports from providers of gaining weight, difficulties with previous diseases, excess of screen time, unhealthy diet and lack of physical exercise (Campos et al., 2022; Ferracioli, & Santos, 2022; Messias & Cury, 2021; Silva et al., 2022).

The conflicts between online work and home tasks were noteworthy: in some studies, Brazilian providers described an overload related to work and home chores, such as taking care of children, cleaning the house, and cooking (Ferracioli, & Santos, 2022; Silva & Ramos, 2020). In a study of 378 Brazilian psychologists, 70% reported moderate changes in their mental health since the onset of pandemic (depression, anxiety, stress, grief, fear), and this emotional distress remained throughout all the phases of COVID-19 pandemic (Campos et al., 2022; Ferracioli, & Santos, 2022; Messias & Cury, 2021; Silva & Ramos, 2020).

To address the personal challenges, providers’ coping strategies included agenda flexibility, self-compassion, peer supervision, study groups, alcohol consumption, and excess of screen time (Campos et al., 2022; Ferracioli, & Santos, 2022). Providers shared the importance of social support, personal psychotherapy, online events, talking to family or friends, enjoying pets, physical exercise, and online movies (Campos et al., 2022; Ferracioli, & Santos, 2022).

Despite the challenges, some Brazilian providers noted advantages, as the increase in access (less evasion and continuity of care), viability for establishing a good interpersonal connection, possibility to assist people from other cities, expansion of professional activities, and affordable prices (Ferracioli, & Santos, 2022; Silva et al., 2022; Vidal et al., 2023). Besides that, they reported the benefits of flexible hours, fewer financial issues, and better time management (Carvalho & Mendes, 2023; Ferracioli, & Santos, 2022; Silva et al., 2022; Vidal et al., 2023).

In Mexico, a sample of family healthcare providers reported that telehealth-based services were effective and safe, providing the opportunity to increase the reach of their services. Further, they expressed a greater flexibility and that it was a practical, economical option (Sanchez-Medina & Rosales-Pina, 2023). Additional positives included the development of tools and resources like the Virtual Psychological Care Guide (Fonseca et al., 2022).

During the COVID-19 pandemic, delivering parenting interventions faced specific barriers, since the challenges outlined could have been enhanced because of the lockdown, hence the importance of understanding providers'perspectives not only about their telehealth services of parenting interventions, but also about how they were providing these services while they themselves were in lockdown (Boldt et al., 2021; Coelho, 2024; Juras et al., 2024). Furthermore, parenting practices suffered changes due to the lockdown, including caregivers´ anxiety and depression, damages in psychosocial well-being, poorer supportive relationships, and work overload, demanding new routines for children, changes in family relationships and adaptations in schooling activities, which could increase the need for parenting guidance (Boldt et al., 2021; Juras et al., 2024; Pokorna et al., 2024).

The international literature shows a paucity of studies on parenting interventions during the COVID-19 pandemic, with research concentrated in China, Europe and USA, focusing mainly on the perception of caregivers, but not on the specific experience reported by the providers (Boldt et al., 2021; Palmer et al., 2023). The studies revealed that parenting intervention online sessions are feasible, acceptable, cost-effective and efficient in moderating the families´ distress, coping strategies, and parent-children relationship (Boldt et al., 2021; Palmer et al., 2023), and sociodemographic factors (except the child's gender) were not associated to the effects of intervention (Pokorna et al., 2024). On the other hand, there were concerns regarding the digital access of population to the online services and the privacy in sessions, besides the lack of studies that approached abuse, maltreatment and violence during social isolation (Boldt et al., 2021).

In the Brazilian context, the protective childhood social network faced major challenges, since the COVID-19 pandemic demanded closing schools, and suspension of in-person support in social assistance centers, which hindered the search for notification and protection regarding the violence against children (Coelho, 2024). Moreover, the difficulties reported by providers were strongly related to establishing effective binding and a secure bond through online sessions: during in-person sessions, usually both parent and child are present, which provides a whole systemic comprehension of family dynamics and follow-up regarding changes in these relationships, but during online sessions only the caregivers were present (Coelho, 2024). Online assistance focused mostly on urgent demands (e.g. the need for immediate food or money due to unemployment), hindering the understanding of complex and continuous difficulties such as intergenerational violence, family distress, and fragile emotional bonds (Coelho, 2024).

On the other hand, some providers detailed that online groups could have helped families to get assisted, since they could not afford to get to the social services, and online meetings would solve this challenge; however, other professionals reported that some families still could not reach support, due to technological difficulties, lack of digital abilities, or the absence of telephone devices or Internet connection (Coelho, 2024).

While there are studies about the perception of providers about their experiences on delivering services through telehealth, not much is known about the challenges experienced by providers delivering parenting interventions during the COVID-19 pandemic in Latinx countries. Additionally, there is a lack of studies in these countries that focus specifically on (1) how providers perceive online guidance related to caregivers and parents; and (2) how providers perceive the positive and negative impact of online assistance in personal and professional context, specifically comparing both countries. Therefore, using a cross-sectional study, the aims of this research were twofold: (1) to understand providers'perspectives about delivering parenting interventions through telehealth during the first wave of the COVID-19 pandemic, comparing Brazil and Mexico; and (2) to examine providers'perspectives about how telehealth and lockdown affected their own personal and professional contexts.

## Methods

The present study includes data collected as part of a larger study that examined the effects of the COVID-19 pandemic on families and mental health providers across the U.S., Brazil, and Mexico. The present study used a subset of the data that was collected during 2020 in Brazil and Mexico during the first and second waves of the pandemic (i.e., First wave March 15th – June 30th, second wave July 1 st – October 15th).

### Sample

The final sample was composed by 111 participants (62 Brazilian and 49 Mexican providers who delivered parenting interventions, including psychologists, mental health counselors, occupational therapists, social workers, and others. The sample was self-selected and embraced providers with diverse experience time and professional contexts (private practice, public health system, community-based organization). Inclusion criteria demanded: being at least 18 years old, and working as a provider who delivered parenting interventions.

### Measures

#### Translations

All measures in the survey were translated from English to Brazilian Portuguese and Spanish, and then back-translated to English to ensure accuracy, according to the World Health Organization guidelines for translation of assessments (World Health Organization, n.d.). Discrepancies were resolved in bi-weekly calls, and the final surveys were approved by all authors. This procedure was necessary due to a lack of available and validated measures in Brazilian Portuguese and Spanish that assessed the constructs relevant to the present study's aims.

#### Demographics

Providers reported information on age, gender, education, marital status, current employment, years of experience, population assisted, and whether they provided in person services during the pandemic.

#### Service delivery

Providers answered six multi-choice items assessing where they worked before the pandemic, population served in-person before the pandemic, current method of conducting therapy, how the population they serve has changed, how content of sessions changed since the pandemic, and how materials for adaptations were obtained. This questionnaire was developed specifically for the present study and the questions are presented with the results in Table 2.

#### Barriers

Providers reported on three items regarding client-level barriers to conducting virtual services (i.e. lack stable internet access or devices, uncomfortable with virtual home visits) (Alpha Spanish = 0.63; Portuguese = 0.32; English = 0.30), and two items related to therapist-level barriers to conducting virtual services (i.e. confidentiality and privacy concerns, difficulty adapting to virtual services) (Alpha Spanish = 0.82; Portuguese = 0.12; English = 0.51). Responses were rated in a Likert scale of: *(*1) not a problem, (2) minor challenge, and (3) major challenge*.* These five sentences were: (1) My clients do not have stable internet access; (2) My clients do not have tablets/webcams, and/or computers; (3) My clients are uncomfortable doing virtual home visits; (4) I am having a hard time adapting my services for virtual visits; (5) I am concerned about confidentiality and privacy.

#### Acceptability, feasibility and appropriateness of telehealth parenting interventions

Providers completed an adapted version of questionnaire about acceptability, feasibility and appropriateness of telehealth parenting interventions (Weiner et al. (2017), a standardized measure in English, translated to Portuguese and Spanish. This section included two items from Acceptability of Intervention Measure subscale—AIM (This new method of meeting is appealing; I like meeting with my clients through this new method), two items from Intervention Appropriateness Measure subscale—IAM (This new method seems fitting; This new method seems suitable), and three items from Feasibility of Intervention Measure subscale—FIM (This new method seems possible; This new method seems doable; This new method seems easy).

Providers reported on items assessing the acceptability (Alpha Spanish = 0.90; Portuguese = 0.93; English = 0.91), appropriateness (Alpha Spanish = 0.80; Portuguese = 0.69; English = 0.69), and feasibility (Alpha Spanish = 0.64; Portuguese = 0.69; English = 0.79) of conducting virtually mediated interventions during the pandemic. Responses were (1) completely disagree, (2) disagree, (3) neither agree nor disagree, (4) agree, and (5) completely agree.

In addition, exploratory items were added specifically for the present study: (1) Is this mode of connection different from before?, (2) How has your work changed during the pandemic in terms of the population that you see?, (3) How has your work changed during the pandemic in terms of the content of the sessions?, (4) Have you looked for resources to support how to provide remote counseling? Which ones?, (5) Regarding the demand from parents to support their children, on a scale of 1 to 5, how much did the challenges for parenting practices increase due to social distancing?, and (6) Do you consider yourself prepared to conduct online sessions?.

*Epidemic-Pandemic Impact Inventory (EPII).* Providers completed the Epidemic–Pandemic Impact Inventory (EPII) (Grasso et al., 2020), regarding the impact of pandemic. The EPII includes 90 statements that indicate potential shifts during the pandemic across some domains (work, home, social functioning, health, and positive changes). Responses were [1] yes (me), [2] yes (person in home), (3) no. Responses were recorded as (1) yes or (0) no.

### Procedures

Survey was administered to participants using Qualtrics, via snowball sampling. The authors sent email and WhatsApp messages to providers from our own professional networks (e.g., REDETAC—https://en.redetac.org/), and groups from academic institutions (eg., Brazilian National Research and Graduate Programs Association https://www.anpepp.org.br/). The invitation is presented in the Appendix, and participants provided informed consent prior to starting the survey. Institutional review board (IRB) approval was obtained from Washington University in Saint Louis on April 22, 2020 (Protocol #202,004,215).

### Data analysis

Responses across language versions were organized by country of residence (i.e., Mexico, Brazil). Information from all available responses were used. Data was analyzed using appropriate inferential statistics depending on whether variables were categorical (i.e., Chi-Square Test of Independence) or continuous (i.e., Kruskal–Wallis Rank Sum Test).

## Results

Table 1 shows demographic and professional data from participants.

**Table 1:** Provider demographics

	Total (*n* = 111)	Brazil (*n* = 62)	Mexico (*n* = 49)	*P*-Value
Gender				.425
Female	99 (89.2%)	53 (85.5%)	46 (93.9%)	
Male	9 (8.1%)	7 (11.3%)	2 (4.1%)	
Prefer not to answer	1 (0.9%)	1 (1.6%)	0 (0%)	
Not listed above:	2 (1.8%)	1 (1.6%)	1 (2%)	
Education				<.001
Some High School	1 (0.9%)	0 (0%)	1 (2%)	
High School	2 (1.8%)	2 (3.2%)	0 (0%)	
Bachelor's Degree	39 (35.1%)	18 (29%)	21 (42.9%)	
Master's Degree	31 (27.9%)	8 (12.9%)	23 (46.9%)	
Ph.D. or higher	9 (8.1%)	6 (9.7%)	3 (6.1%)	
Trade School	28 (25.2%)	28 (45.2%)	0 (0%)	
Occupation				.640
Mental health counselor	3 (2.7%)	2 (3.2%)	1 (2%)	
Occupational therapist	2 (1.8%)	2 (3.2%)	0 (0%)	
Other:	35 (31.5%)	17 (27.4%)	18 (36.7%)	
Psychologist	65 (58.6%)	37 (59.7%)	28 (57.1%)	
Social worker	4 (3.6%)	2 (3.2%)	2 (4.1%)	
Years of experience				.580
0 to 3 years	26 (23.4%)	14 (22.6%)	12 (24.5%)	
4 to 7 years	27 (24.3%)	17 (27.4%)	10 (20.4%)	
8 to 15 years	34 (30.6%)	17 (27.4%)	17 (34.7%)	
16 to 29 years	19 (17.1%)	13 (21%)	6 (12.2%)	
30 years or more	3 (2.7%)	1 (1.6%)	2 (4.1%)	
Working during pandemic				1
Yes	101 (91%)	57 (91.9%)	44 (89.8%)	
No	9 (8.1%)	5 (8.1%)	4 (8.2%)	
Provided in-person services				.715
Yes	40 (36%)	21 (33.9%)	19 (38.8%)	
No	53 (47.7%)	31 (50%)	22 (44.9%)	

Providers from Brazil were more likely to report training in a trade school relative to those from Mexico who most commonly had a masters or bachelors degree. Providers did not differ between countries in gender, occupation, years of experience, whether they were working during the pandemic, and if they continued to provide in-person services (See Table 1).

Providers in Brazil most frequently reported working in private practice, and Mexican providers reported mainly employment in the public health system (See Table 2). Providers in both countries offered several types of services for families, but most commonly offered individual treatment for children and adolescents. The most common methods of delivering services during the pandemic was online telehealth in both countries. Mexican providers also frequently endorsed conducting sessions by phone or Facebook.

**Table 2:** Support service delivery

	Total (*n* = 111)	Brazil (*n* = 62)	Mexico (*n* = 49)
Workplace before pandemic			
Private practice	45 (40.5%)	32 (51.6%)	13 (26.5%)
Health system	37 (33.3%)	13 (21%)	24 (49%)
Community-based organization	11 (9.9%)	4 (6.5%)	7 (14.3%)
Remotely/online	8 (7.2%)	6 (9.7%)	2 (4.1%)
I was unemployed	7 (6.3%)	4 (6.5%)	3 (6.1%)
Other	15 (13.5%)	13 (21%)	2 (4.1%)
Population served in-person before pandemic			
Individual with children and adolescents only	57 (51.4%)	36 (58.1%)	21 (42.9%)
Individual with parents/caregivers only	25 (22.5%)	15 (24.2%)	10 (20.4%)
Family with parents/caregivers and children	38 (34.2%)	26 (41.9%)	12 (24.5%)
Group with children and adolescents only	18 (16.2%)	11 (17.7%)	7 (14.3%)
Group with parents/caregivers only	9 (8.1%)	2 (3.2%)	7 (14.3%)
Group with parents/caregivers and children	15 (13.5%)	5 (8.1%)	10 (20.4%)
Other	23 (20.7%)	14 (22.6%)	9 (18.4%)
Current method of conducting sessions			
Online (e.g., zoom, webex)	73 (65.8%)	42 (67.7%)	31 (63.3%)
By phone	36 (32.4%)	15 (24.2%)	21 (42.9%)
Instagram	5 (4.5%)	2 (3.2%)	3 (6.1%)
Facebook	16 (14.4%)	3 (4.8%)	13 (26.5%)
Other	13 (11.7%)	7 (11.3%)	6 (12.2%)
How population seen has changed since pandemic			
Only adults/parents and now parents/children	6 (5.4%)	3 (4.8%)	3 (6.1%)
Only children and now only parents	18 (16.2%)	11 (17.7%)	7 (14.3%)
Only children and now parents/children	11 (9.9%)	7 (11.3%)	4 (8.2%)
Nothing changed in terms of the population	57 (51.4%)	33 (53.2%)	24 (49%)
How has content of sessions changed since pandemic			
Adapted materials for parenting during social distancing	54 (48.6%)	32 (51.6%)	22 (44.9%)
Adapted to incorporate materials about COVID-19	37 (33.3%)	20 (32.3%)	17 (34.7%)
No change	17 (15.3%)	12 (19.4%)	5 (10.2%)
Other	9 (8.1%)	5 (8.1%)	4 (8.2%)
Obtained materials			
In videos	39 (35.1%)	20 (32.3%)	19 (38.8%)
From workshops or trainings	31 (27.9%)	19 (30.6%)	12 (24.5%)
Live-sessions on social media	38 (34.2%)	22 (35.5%)	16 (32.7%)
Supervision	22 (19.8%)	13 (21%)	9 (18.4%)
Peer-support/peer-mentoring	32 (28.8%)	23 (37.1%)	9 (18.4%)
Searched for research and scientific articles	28 (25.2%)	15 (24.2%)	13 (26.5%)
Created own guidance materials, manuals, booklets	25 (22.5%)	16 (25.8%)	9 (18.4%)
Other	2 (1.8%)	2 (3.2%)	0 (0%)

Most providers reported that their clients’ population did not change during the pandemic. Providers in both countries reported needing to adapt session materials for implementing parenting practice interventions during social distancing. Materials for interventions implemented during the pandemic were obtained from a variety of sources in each country, the most common being videos, live-sessions on social media, and peer-support or peer-mentoring.

Providers did not significantly differ in client barriers engaging in virtual services (See Table 3). Providers in Mexico reported less severe barriers to conducting technology mediated therapy sessions. Specifically, providers in Mexico reported fewer challenges with adapting to virtual services relative to those in Brazil. Providers did not significantly differ in their perceived acceptability and appropriateness of virtual services with youth and families. Providers in both countries did not differ in challenges experienced with implementing parting practices and conducting therapy online during the pandemic.

**Table 3:** Barriers and facilitators

	Total (*n* = 111)	Brazil (*n* = 62)	Mexico (*n* = 49)	*P*-Value
**Client barriers**	1.86 (0.51)	1.85 (0.51)	1.88 (0.51)	.784
Lack stable internet access				.240
Not a problem	24 (21.6%)	17 (27.4%)	7 (14.3%)	
Minor challenge	33 (29.7%)	16 (25.8%)	17 (34.7%)	
Major challenge	17 (15.3%)	10 (16.1%)	7 (14.3%)	
No tablets/webcams, and/or computers				.436
Not a problem	30 (27%)	20 (32.3%)	10 (20.4%)	
Minor challenge	25 (22.5%)	13 (21%)	12 (24.5%)	
Major challenge	14 (12.6%)	7 (11.3%)	7 (14.3%)	
Uncomfortable with virtual home visits				.066
Not a problem	21 (18.9%)	9 (14.5%)	12 (24.5%)	
Minor challenge	30 (27%)	17 (27.4%)	13 (26.5%)	
Major challenge	19 (17.1%)	15 (24.2%)	4 (8.2%)	
**Therapist barriers**	1.96 (0.62)	2.15 (0.56)	1.72 (0.61)	.003
Difficulty adapting to virtual services				.008
Not a problem	21 (18.9%)	7 (11.3%)	14 (28.6%)	
Minor challenge	36 (32.4%)	22 (35.5%)	14 (28.6%)	
Major challenge	14 (12.6%)	12 (19.4%)	2 (4.1%)	
Confidentiality and privacy concerns				.192
Not a problem	23 (20.7%)	11 (17.7%)	12 (24.5%)	
Minor challenge	23 (20.7%)	12 (19.4%)	11 (22.4%)	
Major challenge	25 (22.5%)	18 (29%)	7 (14.3%)	
**Service implementation**				
Acceptability	3.24 (1.12)	3.06 (1.11)	3.48 (1.10)	.107
Appropriateness	3.38 (0.99)	3.22 (0.99)	3.58 (0.97)	.126
Feasibility	3.57 (0.83)	3.38 (0.83)	3.81 (0.78)	.029
**COVID-19 Challenges**				
Difficulties with implementing parenting practices	3.70 (1.19)	3.93 (1.14)	3.39 (1.20)	.052
Preparation to conduct online therapy	3.51 (1.00)	3.55 (0.99)	3.45 (1.03)	.689

Providers across countries experienced 4.58 COVID-19 related challenges in their work (See Table 4). Providers did not significantly differ in number or specific challenges experienced between countries. The most commonly endorsed challenges were having reduced work hours or being furloughed and increases in workload or work responsibilities.

**Table 4:** COVID-19 changes to work

	Total (*n* = 111)	Brazil (*n* = 62)	Mexico (*n* = 49)	*P*-Value
**Number of work challenges**	4.58 (2.95)	4.09 (2.17)	5.70 (4.16)	.271
Laid off from job or had to close own business				.804
Yes	15 (13.5%)	7 (11.3%)	8 (16.3%)	
No	40 (36%)	22 (35.5%)	18 (36.7%)	
Reduced work hours or furloughed				.665
Yes	42 (37.8%)	25 (40.3%)	17 (34.7%)	
No	20 (18%)	10 (16.1%)	10 (20.4%)	
Had to lay-off or furlough employees or people supervised				.961
Yes	15 (13.5%)	9 (14.5%)	6 (12.2%)	
No	32 (28.8%)	21 (33.9%)	11 (22.4%)	
Had to continue to work even though in close contact with people who might be infected				.725
Yes	34 (30.6%)	21 (33.9%)	13 (26.5%)	
No	26 (23.4%)	14 (22.6%)	12 (24.5%)	
Spend a lot of time disinfecting at home due to close contact with people who might be infected at work				.616
Yes	27 (24.3%)	14 (22.6%)	13 (26.5%)	
No	29 (26.1%)	18 (29%)	11 (22.4%)	
Increase in workload or work responsibilities				.080
Yes	38 (34.2%)	17 (27.4%)	21 (42.9%)	
No	24 (21.6%)	17 (27.4%)	7 (14.3%)	
Hard time doing job well because of needing to take care of people in the home				.086
Yes	25 (22.5%)	11 (17.7%)	14 (28.6%)	
No	25 (22.5%)	18 (29%)	7 (14.3%)	
Hard time making the transition to working from home				.798
Yes	34 (30.6%)	20 (32.3%)	14 (28.6%)	
No	25 (22.5%)	13 (21%)	12 (24.5%)	
Provided direct care to people with the disease				1
Yes	19 (17.1%)	11 (17.7%)	8 (16.3%)	
No	36 (32.4%)	22 (35.5%)	14 (28.6%)	
Provided supportive care to people with the disease				.680
Yes	16 (14.4%)	8 (12.9%)	8 (16.3%)	
No	38 (34.2%)	23 (37.1%)	15 (30.6%)	
Provided care to people who died as a result of the disease				.085
Yes	6 (5.4%)	1 (1.6%)	5 (10.2%)	
No	46 (41.4%)	29 (46.8%)	17 (34.7%)	

## Discussion

### Providers´ perspectives about delivering health assistance through telehealth

Data suggests that providers in both countries delivered services during the pandemic using telehealth, but Mexican providers more frequently endorsed conducting sessions by phone and Facebook. This data is consistent with literature, which highlighted that switching to online assistance included a gradual process with the use of hybrid methods (Campos et al., 2022). A key component of shifting to providing parenting interventions via telehealth is adaptability (Sullivan et al., 2021). Providers need to tailor treatment protocols to the family within the context of their technological realities and engagement needs (Sanchez et al., 2024). The providers in our sample expressed needing to adapt session materials. They sought guidance from videos, live-sessions on social media, and peer-support/peer-mentoring, and needed to modify approaches and leaning on the aforementioned tools for guidance fits with similar scenarios described in the literature (Campos et al., 2022; Ferracioli, & Santos, 2022).

The literature about providers’ perspectives on delivering behavioral parenting interventions via telehealth is sparse (Boldt et al., 2021). Providers expressed mixed reactions to the shift to telehealth, since they noted that the frequency and duration of sessions decreased, the time spent interacting with the child directly decreased, and that children's progress and family engagement was mixed (Bravo et al., 2023; Coelho, 2024). The sample of our providers generally found that telehealth was moderately acceptable, appropriate, and feasible, and noted concerns particularly with the need to adapt their approach and ensure client confidentiality. While there are benefits to providing telehealth-based services (Sullivan et al., 2021; Sanchez et al., 2024), our findings suggest that providers view there are trade offs.

Considering the challenges perceived by providers, the sample did not significantly differ in client barriers to engaging in virtual services, but providers in Mexico reported less severe challenges to conducting technology mediated sessions when compared to those in Brazil. Near half of the overall sample reported that the internet access from clients or not having tablets or computers was a minor/major challenge, which is in discordance with previous literature (Caetano et al., 2020; Carvalho & Mendes, 2023; Santos et al., 2023; Silva et al., 2022; Vidal et al., 2023). Nearly half of the overall sample reported confidentiality and privacy concerns, which converges with previous literature (Messias & Cury, 2021; Santos et al., 2023; Silva et al., 2022; Vidal et al., 2023).

### Providers´ perspectives about how lockdown affected personal and professional contexts

The COVID-19 pandemic impacted the personal lives of providers in several ways. Both Brazilian and Mexican samples reported a decrease in self care activities, especially less physical activity, overeating, and more time being sedentary, with a slightly greater imbalance in the Brazilian sample, similar to previous research (Campos et al., 2022; Ferracioli & Santos, 2022; Messias & Cury, 2021; Silva et al., 2022). There was a greater amount of screentime, mental health issues, and sleep problems for both samples (Campos et al., 2022; Ferracioli, & Santos, 2022; Messias & Cury, 2021; Silva & Ramos, 2020) (see Table 5 in Appendix).

Overall, Brazilian providers did not mention problems related to their children’s behavior, but Mexican participants reported their children's emotional issues and sleep disturbance. Other researchers have found similar patterns (Juras et al., 2024; Marques et al., 2020; Requenes et al., 2023; Rodrigues de la Iglesia, 2020; Roos et al., 2021). Brazilian providers mentioned less economic impact when compared to Mexican participants, although the Brazilian literature highlighted some decrease in income (Campos et al., 2022; Messias & Cury, 2021) (see Table 5 in Appendix).

Concerning the impacts at home, the Brazilian sample reported less difficulties related to the family relationships (verbal or physical conflicts, overload in child care), when compared to the Mexican sample, which was inconsistent with previous research (Campos et al., 2022; Ferracioli, & Santos, 2022; Silva & Ramos, 2020). The impact on social activities due to social isolation was a reality for participants in both countries (see Table 6 in Appendix).

Considering the coping strategies, social support was reported by participants in both countries (but specially by Brazilian participants), aligned with recent literature; however, the use of alcohol was not reported, inconsistent with the previous literature (Campos et al., 2022; Ferracioli, & Santos, 2022) (see Table 6 in Appendix).

Participants also reported positive impacts, especially quality time and improved relationships with family*/*friends, increase in physical activity, new hobbies or enjoyable activities, and greater meaning in work, data consistent with Brazilian literature (Ferracioli, & Santos, 2022). The relationship with family or friends and new connections with supportive people were mainly reported by the Brazilian sample, and can be understood as a coping strategy. However, there was a slightly greater balance between *yes/no* answers in the Mexican sample, and Brazilian providers reported more positive impacts (see Table 7 in Appendix).

The COVID-19 pandemic also impacted the providers professionally. Providers from both countries experienced challenges in their work, without significant differences between Brazilian and Mexican samples. The most commonly endorsed challenges were having reduced work hours, which fits with findings from other studies (Campos et al., 2022; Messias & Cury, 2021). Other examples included being furloughed, increases in workload, and increases in work/home responsibilities (Campos et al., 2022; Ferracioli & Santos, 2022; Silva & Ramos, 2020).

### Limitations

There were limitations regarding this study, particularly related to diminished sample size and missing responses, which impacted the statistical rigor and robustness. However, the data presented highlights the similarities and differences faced by providers in their respective countries of origin. Besides that, other demographic data could have been demanded, as the kind of work (the specific and detailed type of parenting interventions that were provided, for how long the providers have been delivering these services, if they changed the formal context of work during the pandemic, etc.), and other details (place of living, number and age of children etc.). These data could have provided more detailed and specific analysis regarding the sample´s personal and professional context, as well as other significant statistical associations.

Future research should use a larger and more representative sample for each country that can be compared based on potentially important professional factors, in addition to comparing other cultures/countries, and include more professional and demographic characteristics. Despite these limitations, this study can provide support for future research in parenting intervention contexts, in addition to important information on cultural differences in Latin-American countries in the context of mental health care, which needs to be expanded.

## Conclusions

This study aimed to compare the perceptions of Brazilian and Mexican providers that worked with parenting practices in adapting to online care, given the challenges imposed by the COVID-19 pandemic. The results showed several difficulties, related to task overload, reduced income, persistent adverse emotions, acquisition of unhealthy habits, feer notifications of violence, and issues related to technology/internet use. On the other hand, the data also pointed out advantages of online care, suggesting that this is a feasible and viable model for assistance.

To improve the experience of telehealth, our findings justify a few suggestions. Treatment developers should offer treatment-concordant adaptations for virtual service delivery: this would help providers know the most effective way to tailor treatment. When providers first engage families, they should verify the family’s access to needed telehealth resources (e.g., internet, computer/tablet, webcams), and the family’s ability to use those tools. Agencies might consider purchasing tablets that families could use for the duration of their services. Providers should also develop guidelines for families to ensure that privacy is maintained during a virtual session (e.g., family should be alone in the room during the session, etc.). Also, it would be urgent that countries develop new public policies that may assure digital inclusion, with a deeper understanding on how bonding and vinculation can be hindered during online sessions.

## Data Availability

The datasets used and/or analyzed during the current study are available from the corresponding author upon reasonable request.

## Appendix

Invitation to participate in the research: We invite you to participate in the present study which aims to evaluate the changes of healthcare delivery during the quarantine period of COVID- 19. If you agree to participate, you will answer some questions about your experience providing support to families/parents/children during the period of quarantine. The duration of this questionnaire will be approximately 30 minutes. We will not obtain any identifying data about you (you will not be asked to disclose your name or any identifying information about yourself). It will not be possible to identify you through your answers. Your participation in this study is voluntary. The data collected with your participation in the study will be recorded and cannot be removed. All data collected in the study is confidential. If you do not want to participate in the study, or decide to end your participation in it, you can do so by closing the survey. If you have any questions, you can reach out to Dr. Ana A. Baumann, email abaumannwalker@wustl.edu. You may contact the Human Research Protection Office at 1- (800)-438-0445 or hrpo@wustl.edu. Thank you very much for your consideration of this research study.

**Table 5:** Impacts of COVID19 in aspects of daily life

	Brazil (*n *= variable)		Mexico (*n* = variable)	
*Impact on physical and health problems*	No	Yes	No	Yes
Increase in health problems not related to this disease.	21(77%)	6(22%)	15(55%)	12(44%)
Less physical activity or exercise.	6(20%)	24(80%)	10(37%)	17(63%)
Overeating or eating more unhealthy foods (e.g., junk food).	9(28%)	23(71%)	12(44%)	15(55%)
More time sitting down or being sedentary.	6(19%)	25(80%)	6(22%)	21(77%)
Important medical procedure canceled (e.g., surgery).	21(95%)	1(%%)	17(73%)	6(26%)
Unable to access medical care for serious conditions (dialysis/chemo)	18(100%)	0(0%)	14(93%)	1(7%)
Got less medical care than usual (routine,preventive care appointments).	9(40%)	13(60%)	12(50%)	12(50%)
Elderly/disabled family member not in home unable to get help they need	21(95%)	1(5%)	13(72%)	5(28%)
*Impact on health and emotional problems*				
Increase in child behavioral or emotional problems.	14(77%)	4(22%)	7(50%)	7(50%)
Increase in child’s sleep difficulties or nightmares	17(94%)	1(6%)	1(14%)	6(86%)
Increase in mental health problems or symptoms (mood, anxiety, stress).	7(23%)	23(76%)	9(34%)	17(65%)
Increase in sleep problems or poor sleep quality.	6(22%)	22(78%)	5(18%)	22(82%)
Increase in use of alcohol or substances.	16(64%)	9(36%)	21(87%)	3(13%)
Unable to access mental health treatment or therapy.	17(70%)	7(30%)	17(68%)	8(32%)
Not satisfied with changes in mental health treatment or therapy.	13(76%)	4(24%)	16(77%)	5(23%)
More time on screens and devices (phone, video games, watching TV).	8(25%)	23(75%)	3(12%)	23(88%)
*Impact on economic issues*				
Unable to get enough food or healthy food	24(93%)	2(7%)	11(44%)	14(56%)
Unable to access clean water	26(96%)	1(4%)	16(64%)	9(36%)
Unable to pay important bills like rent or utilities	25(92%)	2(8%)	13(50%)	13(50%)
Difficulty getting places due to less access to public transportation or concerns about safety	18(65%)	10(35%)	19(80%)	5(20%)
Unable to get needed medications (e.g., prescriptions or over-the-counter)	25(93%)	2(7%)	16(66%)	8(33%)

**Table 6:** Impacts of COVID19 in aspects of home/family life and social activities

	Brazil (*n *= variable)		Mexico (*n* = variable)	
*Impacts on home/family life*	No	Yes	No	Yes
Childcare or babysitting unavailable when needed.	14(70%)	6(30%)	6(50%)	6(50%)
Difficulty taking care of children in the home.	13(68%)	6(32%)	7(44%)	9(56%)
More conflict with child or harsher in disciplining child or children.	15(78%)	4(22%)	9(60%)	6(40%)
Had to take over teaching or instructing a child.	11(62%)	7(38%)	7(47%)	8(53%)
Family or friends had to move into your home.	20(90%)	2(10%)	21(84%)	4(16%)
Had to spend a lot more time taking care of a family member	20(74%)	7(26%)	13(52%)	12(48%)
Had to move or relocate.	21(88%)	3(12%)	25(96%)	1(4%)
Became homeless.	28(100%)	0(0%)	22(92%)	2(8%)
Increase in verbal arguments or conflict with a partner or spouse.	18(65%)	10(35%)	15(68%)	7(32%)
Increase in physical conflict with a partner or spouse.	26(100%)	0(0%)	18(82%)	4(18%)
Increase in verbal arguments or conflict with other adult(s) in home.	23(85%)	4(15%)	17(68%)	8(32%)
Increase in physical conflict with other adult(s) in home.	27(100%)	0(0%)	19(76%)	6(24%)
Increase in physical conflict among children at home.	22(95%)	1(5%)	15(78%)	4(22%)
*Impact on social activities*				
Separated from family or close friends	11(35%)	21(65%)	5(18%)	22(82%)
Did not have ability/resources to talk to family/friends while separated	25(93%)	2(7%)	12(50%)	12(50%)
Unable to visit loved ones in a care facility (nursing home, group home).	16(76%)	5(24%)	9(53%)	8(47%)
Family celebrations canceled or restricted.	4(14%)	26(86%)	2(7%)	25(93%)
Planned travel or vacations canceled.	7(25%)	20(75%)	5(18%)	22(82%)
Religious or spiritual activities canceled or restricted.	5(18%)	22(82%)	3(15%)	17(85%)
Unable to be with a close family member in critical condition.	16(76%)	5(24%)	9(60%)	6(40%)
Unable to attend in-person funeral/religious service (dead family member/friend)	15(72%)	6(28%)	8(47%)	9(53%)
Unable to participate in social clubs, sports teams, or usual volunteer activities.	4(13%)	27(87%)	10(38%)	16(62%)
Unable to do enjoyable activities or hobbies.	7(20%)	27(80%)	4(15%)	23(85%)

**Table 7:** Positive impacts of COVID19

	Brazil (*n *= variable)		Mexico (*n* = variable)	
	No	Yes	No	Yes
More quality time with family or friends in person or from a distance (e.g., on the phone, Email, social media).	5(17%)	25(83%)	12(45%)	15(55%)
More quality time with partner or spouse	10(35%)	18(65%)	18(58%)	13(42%)
More quality time with children	5(27%)	13(73%)	5(34%)	10(66%)
Improved relationships with family or friends.	10(34%)	20(66%)	13(48%)	14(52%)
New connections made with supportive people	11(40%)	16(60%)	14(56%)	11(44%)
Increase in exercise or physical activity.	5(16%)	25(84%)	12(44%)	15(56%)_
More time in nature or being outdoors	10(35%)	18(65%)	10(43%)	13(57%)
More time doing enjoyable activities (e.g., reading books, puzzles).	5(27%)	13(73%)	5(34%)	10(66%)
Developed new hobbies or activities	10(34%)	20(66%)	13(48%)	14(52%)
More appreciative of things usually taken for granted	11(40%)	16(60%)	14(56%)	11(44%)
Paid more attention to personal health	24(75%)	8(25%)	16(62%)	10(38%)
Paid more attention to preventing physical injuries	27(85%)	5(15%)	22(845)	4(16%)
Ate healthier foods.	14(42%)	19(58%)	12(44%)	15(55%)
Less use of alcohol or substances.	5(16%)	25(84%)	12(44%)	15(55%)
Spent less time on screens or devices outside of work hours (e.g., looking at phone, playing video games, watching TV).	10(35%)	18(65%)	10(44%)	13(66%)
Volunteered time to help people in need	5(27%)	13(73%)	5(34%)	10(66%)
Donated time or goods to a cause related to this disease (e.g., made masks, donated blood, volunteered).	10(34%)	20(66%)	13(48%)	14(52%)
Found greater meaning in work, employment, or school	11(40%)	16(60%)	14(56%)	11(44%)
More efficient or productive in work, employment, or school	24(75%)	8(25%)	16(62%)	10(38%)

## Abbreviations

IRB: Institutional review board
AIM: Intervention Measure subscale
IAM: Intervention Appropriateness Measure
FIM: Feasibility of Intervention Measure
EPII: Epidemic–pandemic impact inventory
